# Evaluation of the Consumption of Junk Food Products and Lifestyle among Teenagers and Young Population from Romania

**DOI:** 10.3390/nu16111769

**Published:** 2024-06-05

**Authors:** Magdalena Mititelu, Gabriela Stanciu, Monica Licu, Sorinel Marius Neacșu, Mariana Floricica Călin, Adrian Cosmin Roșca, Tiberius Iustinian Stanciu, Ștefan Sebastian Busnatu, Gabriel Olteanu, Steluța Constanța Boroghină, Teodor Octavian Nicolescu, Felicia Suciu, Carmen Elena Lupu

**Affiliations:** 1 Department of Clinical Laboratory and Food Safety, Faculty of Pharmacy, “Carol Davila” University of Medicine and Pharmacy, 3-6, Traian Vuia Street, Sector 2, 020956 Bucharest, Romania; magdalena.mititelu@umfcd.ro (M.M.); gabriel.olteanu@mst.umfcd.ro (G.O.); 2 Department of Chemistry and Chemical Engineering, “Ovidius” University of Constanta, 900527 Constanta, Romania; gstanciu@univ-ovidius.ro; 3 Department of Ethics and Academic Integrity, Faculty of Medicine, “Carol Davila” University of Medicine and Pharmacy, 050474 Bucharest, Romania; monica.licu@umfcd.ro; 4 Department of Pharmaceutical Technology and Bio-Pharmacy, Faculty of Pharmacy, “Carol Davila” University of Medicine and Pharmacy, 020945 Bucharest, Romania; sorinel-marius.neacsu@drd.umfcd.ro; 5 Faculty Psychology & Educational Sciences, “Ovidius” University of Constanta, 900527 Constanta, Romania; mariana.calin@365.univ-ovidius.ro; 6 Department of Drug Analysis, Biopharmacy and Biological Medicines, Faculty of Pharmacy, “Ovidius” University of Constanta, 900470 Constanta, Romania; felicia.suciu@365.univ-ovidius.ro; 7 Press Office, “Ovidius” University of Constanta, 900527 Constanta, Romania; 8 Department of Cardio-Thoracic Pathology, Faculty of Medicine, “Carol Davila” University of Medicine and Pharmacy, 050474 Bucharest, Romania; stefan.busnatu@umfcd.ro; 9 Department of Complementary Sciences, History of Medicine and Medical Culture, Faculty of Medicine, “Carol Davila” University of Medicine and Pharmacy, 050474 Bucharest, Romania; steluta.boroghina@umfcd.ro; 10 Department of Organic Chemistry, Faculty of Pharmacy, “Carol Davila” University of Medicine and Pharmacy, 020956 Bucharest, Romania; teodor.nicolescu@umfcd.ro; 11 Department of Mathematics and Informatics, Faculty of Pharmacy, “Ovidius” University of Constanta, 900001 Constanta, Romania; clupu@univ-ovidius.ro

**Keywords:** fast food, sweetened beverages, energy drinks, physical activity, metabolic syndrome, insulin resistance

## Abstract

Background: The long-term consumption of junk food products can lead to nutritional and metabolic imbalances, especially when it is associated with a lack of physical activity and the consumption of alcohol or other high-calorie products. Methods: The evaluation of junk food consumption among teenagers and young people in Romania was carried out with the help of a cross-sectional study based on a questionnaire. Results: A total number of 1017 respondents participated in this study, comprising 470 males and 547 females aged between 16 and 25 years. Although the majority of young people fell into the normal-weight category (607 of them, *p* < 0.0001), some aspects can be noted that in the long term can produce a series of nutritional imbalances: an increased tendency toward sedentarism, with 553 (*p* = 0.613) of the respondents declaring that they performed sports rarely or not at all, and a tendency toward relatively high consumption of foods high in calories (fast food products and especially fried potatoes, hamburgers, shawarma, pastries, and snacks, along with sweetened drinks and even alcoholic beverages). The respondents participating in this study even indicated a perceived addiction to the consumption of certain products: coffee (50.48%), fried potatoes (38.9%), hamburgers (37.05%), shawarma (31.65%), and snacks (30.08%). Many of these products are rich in calories, saturated fat, and even trans fat. Conclusions: This study highlights a series of aspects that can have long-term negative effects related to the excess weight associated with other imbalances: consumption preferences among young people for hypercaloric fast food products, sweetened drinks associated with reduced physical activity, and even the development of some forms of food addictions for a series of hypercaloric foods.

## 1. Introduction

Research conducted by the World Health Organization (WHO) and studies featured in esteemed medical publications, like *The Lancet*, indicate a marked surge in the ingestion of unhealthy food among teenagers on a global scale in recent decades [1,2,3,4]. This escalating pattern predominantly manifests in urban settings and nations experiencing rapid economic growth. The consumption of such food items by adolescents is influenced by multifaceted factors, encompassing the accessibility of fast food outlets, convenience stores, and prominently promoted processed food items. Additionally, social elements and peer influence significantly impact dietary preferences [5,6,7,8,9,10]. Moreover, the aggressive marketing of junk food tailored to a younger audience, coupled with increasingly hectic lifestyles that prompt a reliance on fast and expedient, yet often nutritionally deficient food choices, further contribute to this trend [11,12,13,14].

Though precise statistical data fluctuate across regions and countries, the rising prevalence of adolescent consumption of junk food is a global apprehension due to its correlation with diverse health complications, including but not limited to obesity, diabetes, cardiovascular ailments, and other lifestyle-related diseases [15,16,17].

Mitigating measures aimed at addressing this concern encompass educational initiatives designed to advocate healthy eating habits, policies aimed at regulating the marketing of food products to children, and endeavors aimed at enhancing the availability of nutritious food options within educational institutions and local communities [18,19,20].

Different demographic groups exhibit varying propensities for consuming junk food, with some segments displaying more frequent consumption. Among these, adolescents and young adults emerge as predominant consumers of such food items [21,22]. Their dietary preferences are shaped by factors including peer influence, prevailing social patterns, and the allure of easily accessible and fast food alternatives. Moreover, younger children might also partake in junk food consumption due to its widespread marketing and availability, although their choices are typically influenced by parental guidance and decisions. Nonetheless, research suggests that adults, especially individuals aged from their late twenties to mid-forties, also demonstrate considerable consumption of junk food owing to demanding lifestyles, reliance on convenience, and established dietary habits from earlier stages of life. Hence, while adolescents and young adults are commonly spotlighted as primary consumers, the appeal and ingestion of junk food extend across various age brackets [23,24].

Junk food items typically exhibit distinctive characteristics that set them apart from nutritious alternatives, often characterized by elevated levels of refined sugars; unhealthy fats encompassing saturated and trans fats; excessive salt content; and a dearth of essential nutrients such as vitamins, minerals, and fiber. This category of food tends to be low in nutritional quality while bearing a high-calorie load [25,26,27].

The consumption of junk food can have a substantial impact on the health of individuals. This impact encompasses several repercussions, including the potential for weight gain and obesity due to the habitual intake of calorie-dense yet nutrient-deficient junk food [28,29]. Furthermore, the presence of unhealthy fats and excessive sodium in these food items heightens the vulnerability to heart disease, hypertension, and related cardiovascular complications. Additionally, the heightened sugar content prevalent in many junk food products can foster insulin resistance, thereby contributing to the onset of type 2 diabetes. Oral health is also at risk as sugary and acidic junk food can prompt dental cavities and erosion, leading to related dental issues [30,31].

Increased long-term consumption of junk food products can lead to malnutrition, which can be simple or complex depending on the nutritional deficiency of these categories of food products and is usually hypercaloric. Moreover, empirical studies suggest a plausible association between an excessive consumption of junk food and an augmented susceptibility to mental health conditions like depression and anxiety [32,33,34,35].

The adverse health effects of junk food go beyond their composition and extend to the displacement of essential nutrients from healthier dietary sources such as fruits, vegetables, whole grains, and lean proteins. Consequently, the cumulative impact of regular consumption of junk food can markedly compromise an individual’s overall health and well-being, heightening the likelihood of various chronic diseases and health complications [36,37].

There are other aspects that must be considered regarding the safety of consumption of junk food products; besides the abundance of additives, some products may have toxic compounds resulting from excessive thermal processing or the excessive use of frying oil (burgers, fries, etc.) [38,39,40,41]. The quality of the raw material is also very important. The presence of toxic contaminants in the environment (pesticides, microplastics, heavy metals, etc.) can endanger the health and even the life of the consumer [42,43,44,45,46].

Junk food encompasses a wide array of products that tend to be high in calories, sugars, unhealthy fats, and sodium while lacking substantial nutritional value. These can include fast food (burgers, fries, pizzas, and other quick-service meals), sugary beverages (soft drinks, energy drinks, sweetened juices, and flavored waters), snack foods (chips, crisps, candy, chocolates, and other high-calorie snacks), processed foods (ready-to-eat meals; packaged snacks; and convenience foods high in preservatives, added sugars, and unhealthy fat), and baked goods (pastries, cakes, cookies, and sweet baked items).

According to the latest official reports published by the National Institute of Public Health in Romania, the number of new cases of obesity diagnosed in children under the age of 19 registered in 2020 was 6731, representing 18.9% of the total number of cases in the country. A higher incidence was found in girls and much higher in urban compared to rural areas [47]. The adult obesity rate in Romania is around 10%. In 2018, the rate of overweight and obesity among 15-year-old children in Romania was 21%, higher values compared to the European Union average (19%) [48]. According to the World Obesity Atlas 2023, the growth rate of obesity in Romania until 2035 will be 2.1%/year for adults and 5.6%/year for children, a fact that will place Romania among the countries with the most increasing rate of obesity in children [49].

Prompted by the dangers hidden in the excessive consumption of junk food products, a cross-sectional study was carried out based on a questionnaire that evaluates the consumption of junk food products among teenagers and young people from Romania.

## 2. Materials and Methods

### 2.1. Study Design

To assess the consumption of junk food, a validated questionnaire [50] was disseminated, which evaluated the frequency of consumption of junk food products (hamburgers, hot dogs, french fries, shawarma, packed sandwiches, chips, snacks, patisserie products, packaged cakes, candy, ice cream, other packaged sweet products, chewing gum, sweetened carbonated drinks, sweetened non-carbonated drinks, energy drinks, and coffee) but also aspects related to tobacco consumption, physical activity, consumption of alcoholic beverages, and product category food that predominates in the daily diet, along with information related to sex, age, height, weight, and area of residence. Based on the anthropometric data, the body mass index (BMI) was calculated [51]. The Google Forms platform was used to disseminate and centralize the answers to the questionnaire, and the questionnaire was disseminated among high school students during their final classes under the supervision of teachers and with the agreement of the management and parents. Only teenagers between the ages of 16 and 18 completed the questionnaire. For the 19–25 age group, the questionnaire was distributed among students from various universities in Romania. Participation in the study involved obtaining informed consent from the respondents, the protection of personal data, and the preservation of anonymity. The inclusion criteria for the study were being between 16 and 25 years old; parental consent for young people under 18; and being a resident in Romania. The study was conducted in accordance with the Declaration of Helsinki and approved by the Ethics Commission of the Carol Davila University of Medicine and Pharmacy from Bucharest, no. 4201/16.02.2024. The questionnaire was distributed between February 20 and 20 March 2024.

The questionnaire (Supplementary Materials) was uploaded on the Google Forms platform because it offers a number of important advantages: It provides options for setting validation rules to ensure the accuracy and consistency of responses; responses are automatically collected and stored in Google Sheets, allowing for easy analysis and data manipulation and viewing responses in real time, which is particularly useful for events or situations where immediate feedback is required; and it provides strong security measures to protect data, including encryption and secure access controls. Users can also set forms to restrict access to specific groups or individuals, and the questionnaire can be easily shared via email, links, embedded in websites, or through social media, ensuring broad dissemination.

The design of the questionnaire with the help of the Google Forms platform also provided a series of security measures to protect personal data (blocking the collection of addresses and identification data of respondents), thus blocking the collection of multiple answers from the same user. In order to avoid errors related to the age of the respondents, the answers from all the respondents who accessed the questionnaire were collected in the platform, but only the answers of those who fell into the age range of 16–25 years were used for data processing. Thus, out of the 1310 responses recorded, only 1017 valid responses were retained that fell into the age group followed in this study. Collaboration agreements were made with schools, universities, and multinational companies chosen in such a way as to cover the main regions on the territory of Romania, and the adolescents under 18 years completed the questionnaire after being previously instructed by the teaching staff or parents, where they were also made aware of the fact that their identities were protected (the answers were protected so that the identification data of the respondents were not revealed), and they were invited to answer as honestly as possible. The aspects related to the protection of the respondent’s identity and the blocking of multiple answers were also mentioned in the form regarding the terms and conditions of participation in the study. Also, in the consent form, the study participants were informed that the answers would be processed and published, and the respondents were invited to answer as correctly as possible in order not to change the accuracy of the data because their identity was protected.

### 2.2. Statistical Analysis

First, descriptive statistics were used to present the characteristics of participants. Categorical variables were presented with absolute frequencies (n) and relative frequencies (%).

The potential associations between preferred non-alcoholic drinks and junk food consumption with anthropometric data (gender and BMI) were identified by using a chi-square test.

To visualize the relationships between BMI and participant responses regarding the consumption of non-alcoholic beverages and junk food, correspondence analysis (CA) was performed.

Correspondence analysis was performed using XLSTAT (version 2020, Addinsoft, New York, NY, USA). Statistical Package for Social Science, version 23 (SPSS Inc., Chicago, IL, USA), was used for descriptive statistics, and a chi-square test was used to validate the questionnaire and ensure its reliability; *p*-values less than 0.05 were considered statistically significant [52].

In terms of the representativeness of the sample of respondents, according to the last census, the population of Romania between 16 and 25 years old is 1,959,584, representing 10.28% of the total population [53]. The minimum required sample size for our study, calculated using the Cochran formula [54] for a known population size, was 659 (for a 96% confidence interval and ±4% error). The collection of answers from respondents distributed throughout Romania was also considered.

## 3. Results

### 3.1. Socio-Demographic Characteristics of the Respondents

Table 1 presents the socio-demographic characteristics of the respondent groups participating in this study, along with a series of aspects related to physical activity and the consumption of alcoholic beverages. Most of the participants in this study were teenagers aged up to 18 (60%, *p* < 0.028) and from urban areas (81.3%, *p* = 0.002). More than 54.3% (*p* = 0.613) of the respondents rarely performed sports or not at all, and 76.4% (*p* = 0.590) declared that they consume alcoholic beverages 2–3 times a week. Regarding body weight, 59.7% (*p* < 0.0001) were normal weight.

### 3.2. Evaluation of Junk Food Product Consumption

**Figure 1:**
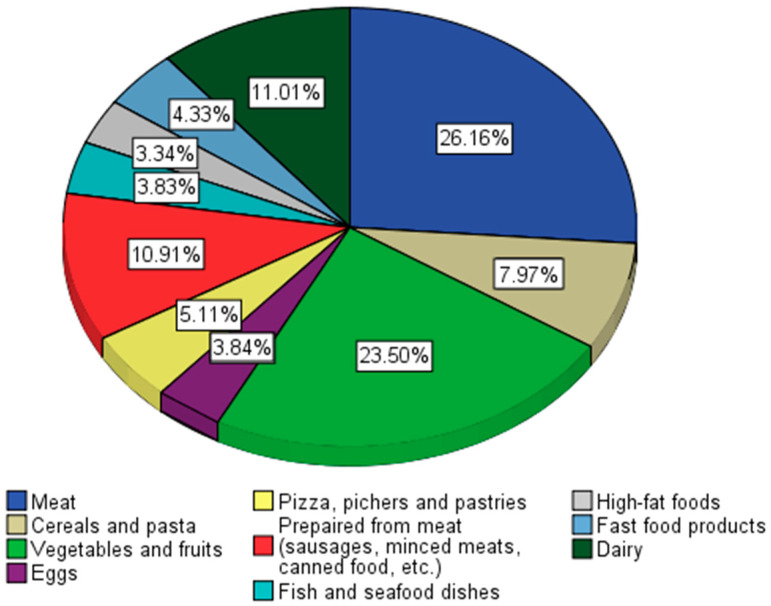
Food categories that predominate in the daily diet.

Among the categories of food products that predominate in the daily diet are meat (26.16%), meat preparations (10.91%), vegetable products, and dairy products (Figure 1). Among the junk food products, the most consumed are pizza and pastries.

**Figure 2:**
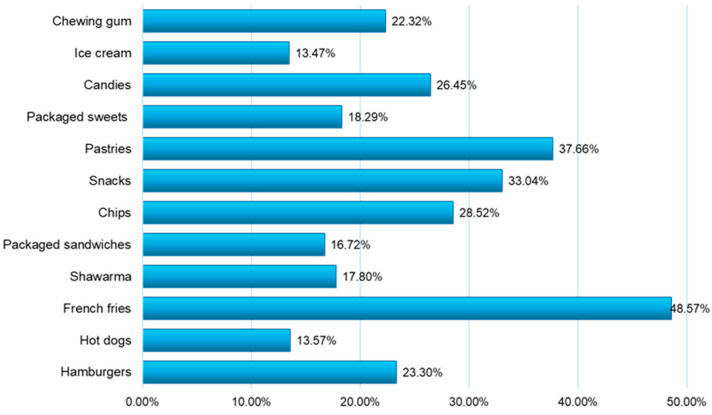
The categories of junk food products indicated in the consumption preferences of young people.

According to the recorded answers, among the most preferred junk food products by young people were fried potatoes (48.57%), pastries, snacks, chips, sweets, and hamburgers (Figure 2).

**Figure 3:**
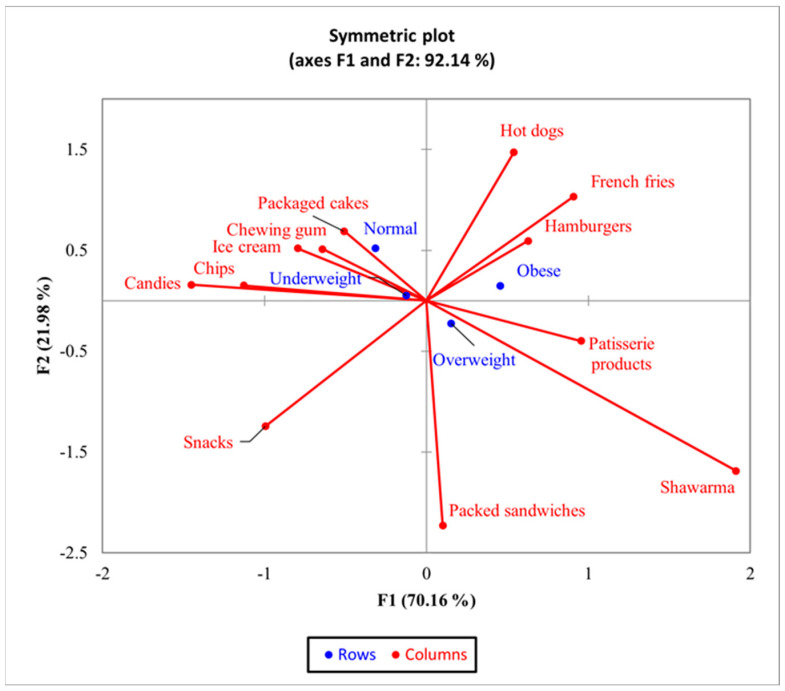
The first two dimensions of correspondence analysis (CA) symmetric plot for BMI groups and all the 12 types of junk food products.

The bi-plot indicated that for the BMI group, 92.14% of the observed variability could be attributed to the two main components (F1: 70.16% and F2: 21.98%). CA showed a significant difference (χ^2^ = 57.42 and *p* = 0.0051) between BMI and the consumption of the 12 types of junk food products (Figure 3) but not between the frequency of consumption of the analyzed products and respondents with normal BMI and the underweight group. There was an increased tendency for consumption of french fries, hamburgers, and hot dogs among obese respondents.

**Figure 4:**
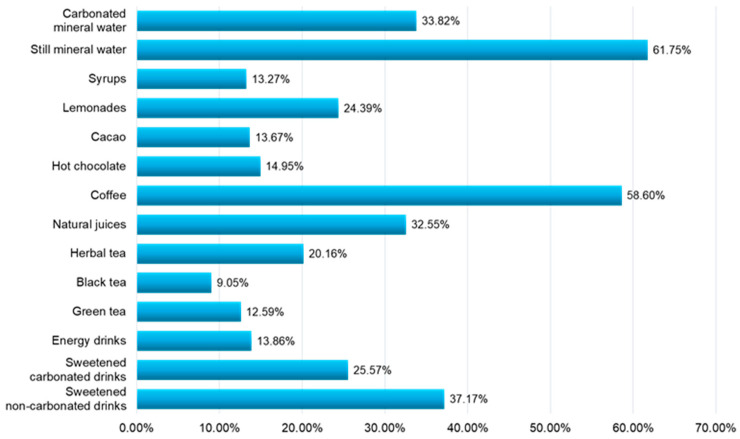
The categories of non-alcoholic beverages indicated in the consumption preferences of young people.

Coffee (58.6%) and sweetened drinks (37.17%) were two of the top non-alcoholic beverage consumption preferences (Figure 4) among the young participants in this study.

**Figure 5:**
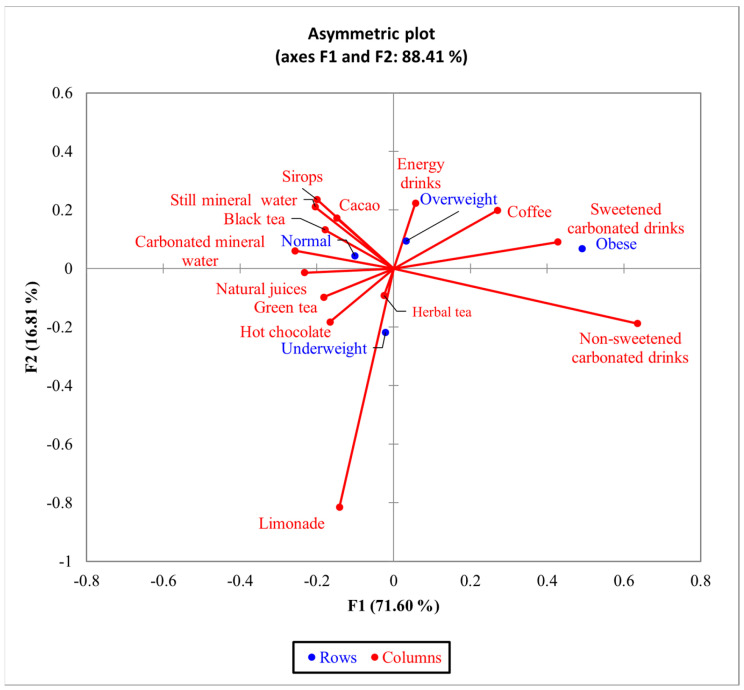
The first two dimensions of correspondence analysis (CA) symmetric plot for BMI groups and all the 14 types of non-alcoholic drinks analyzed.

For BMI, the bi-plot indicated that 88.41% of the variability observed could be attributed to the two principal components for F1 (71.60%) and F2 (16.81%). The CA showed a significant difference (χ^2^ = 52.47 and *p* < 0.0072) between BMI and the 14 types of non-alcoholic beverages (Figure 5). Obese and overweight respondents presented similar profiles related to the consumption of non-alcoholic beverages. There was an increased tendency to consume sweetened carbonated drinks and energy drinks among obese and overweight people.

**Figure 6:**
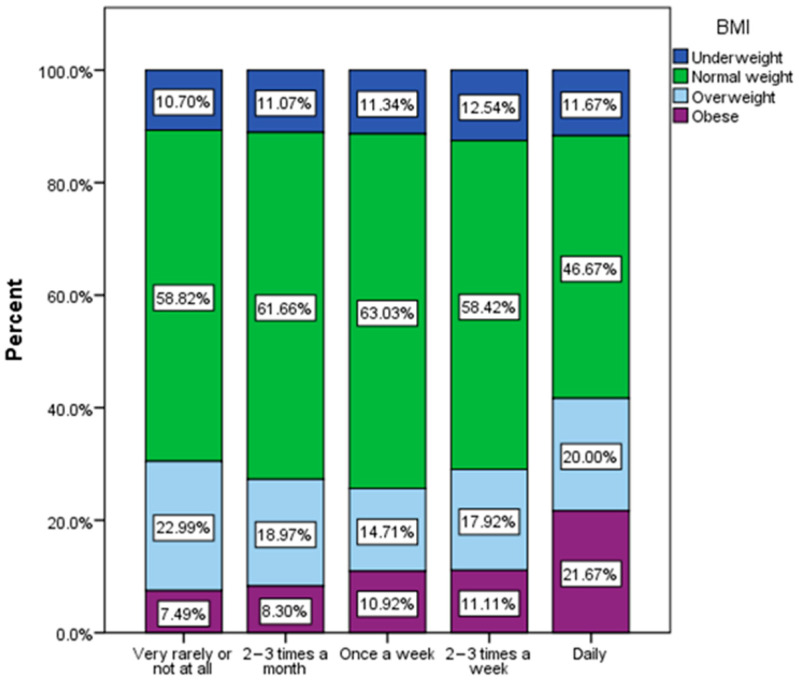
Frequency of junk food product consumption by BMI (χ^2^ = 27.06, *p* = 0.025).

There was a tendency for an increase in the frequency of consumption of junk food products among overweight and especially obese respondents (Figure 6).

**Figure 7:**
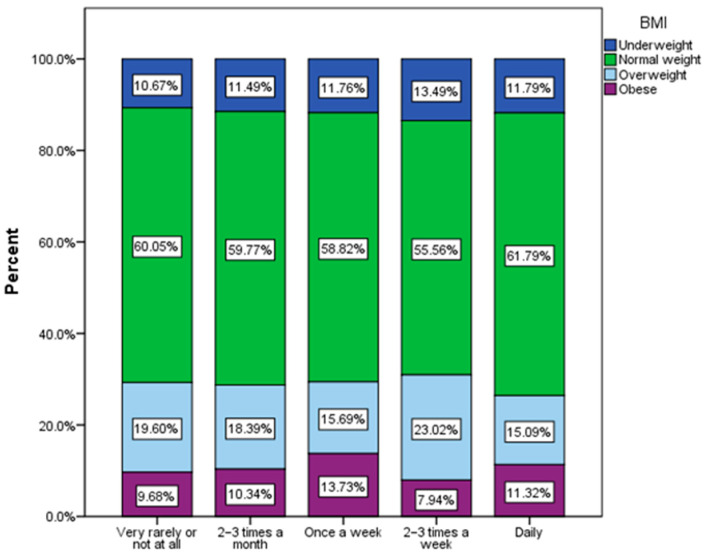
Frequency of non-alcoholic carbonate sweetened beverage consumption by BMI (χ^2^ = 22.78, *p* = 0.030).

Regarding the frequency of consumption of sweetened carbonated non-alcoholic beverages, a tendency toward an increase in the frequency of consumption among normal-weight respondents can be noted (Figure 7).

**Figure 8:**
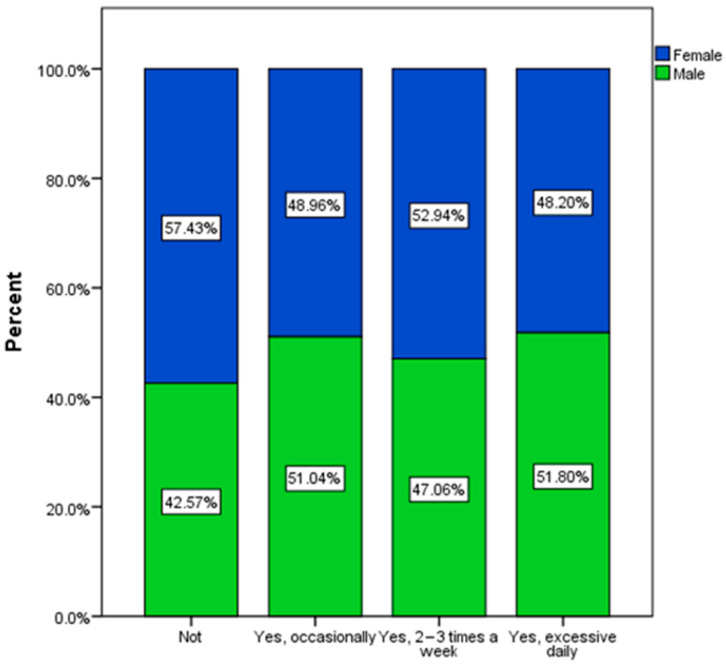
Smoke habits by gender.

Regarding the habit of smoking, there was a higher tendency among male respondents (Figure 8).

**Figure 9:**
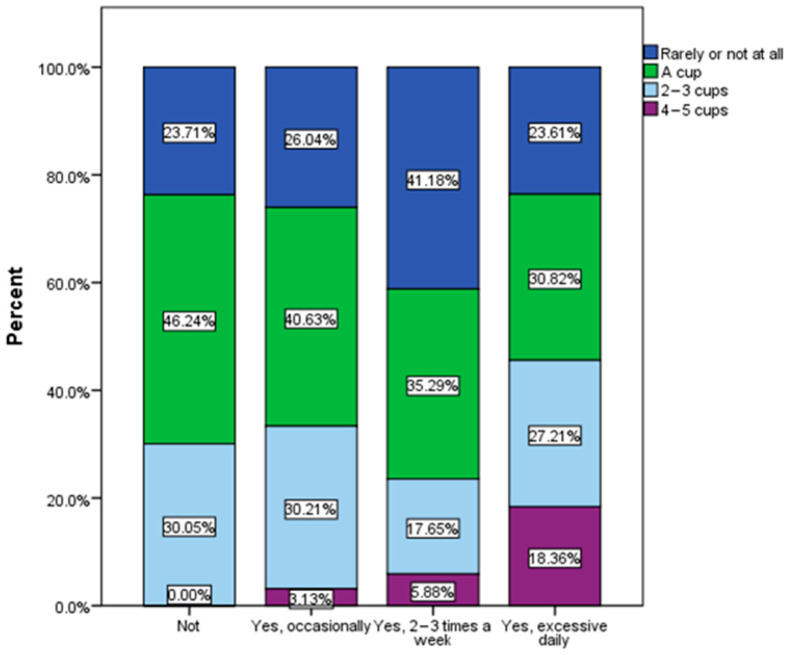
Smoke habits by the amount of coffee consumption (χ^2^ = 78.32, *p* < 0.001).

In general, the increased tendency of coffee consumption was also associated with the habit of smoking (Figure 9).

**Figure 10:**
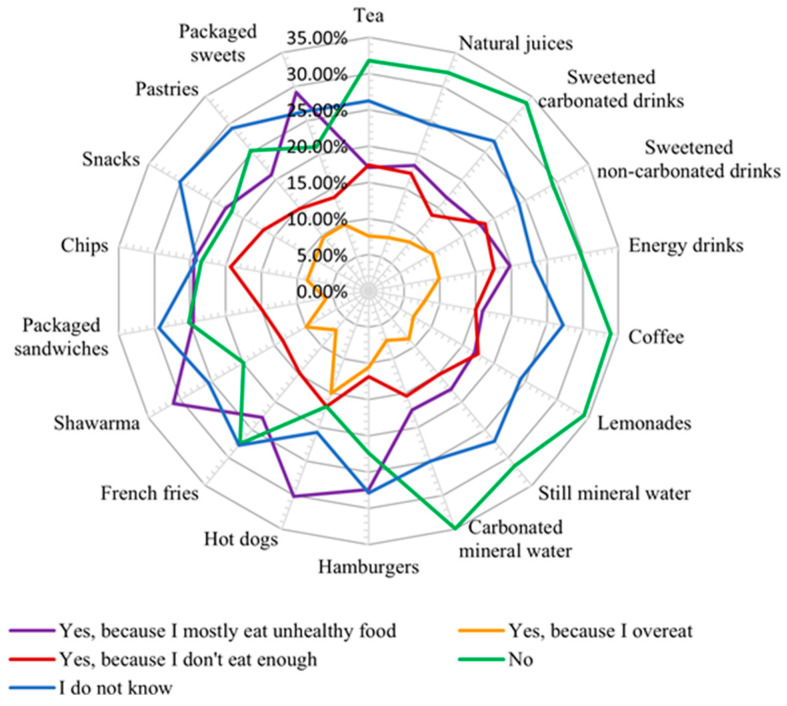
Respondents’ perception of the impact of food on their state of health.

In this study, the respondents were asked to assess whether their health status was negatively influenced by food. Thus, 27.58% stated that they did not consider that food affected their health, but in Figure 10, it can be seen that this group did not excessively consume junk food products and were more oriented toward natural drinks. Notably, 22.19% considered food to negatively influence their health, and these respondents were the ones who mainly consumed junk food products and realized that this type of food was not nutritious. In addition, 28.22% declared that they did not know if food negatively influenced their health, and the rest believed that food influenced their state of health due to excessive or insufficient consumption.

**Figure 11:**
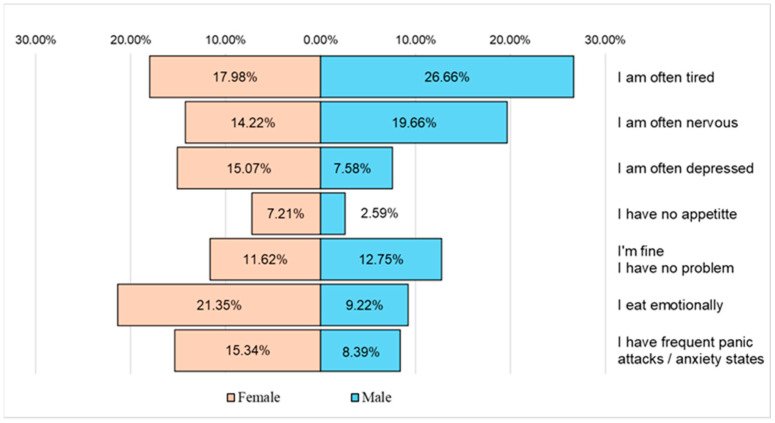
The main problems affecting the respondents’ quality of life.

According to the recorded answers, the respondents frequently faced states of fatigue and nervousness, and the female respondents also indicated problems related to emotional eating and states of depression, panic, or anxiety (Figure 11).

**Figure 12:**
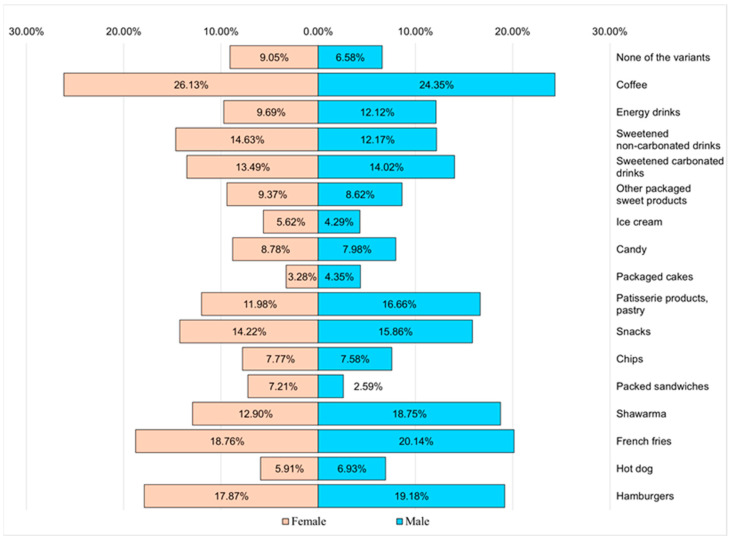
The perception of food addiction among the respondents.

Regarding the respondents’ perception related to the possible development of consumption addictions, it was found that the most incriminated products were coffee, fried potatoes, hamburgers, shawarma, sweetened drinks, snacks, and pastry products (Figure 12). There was a slight difference between the sexes.

**Figure 13:**
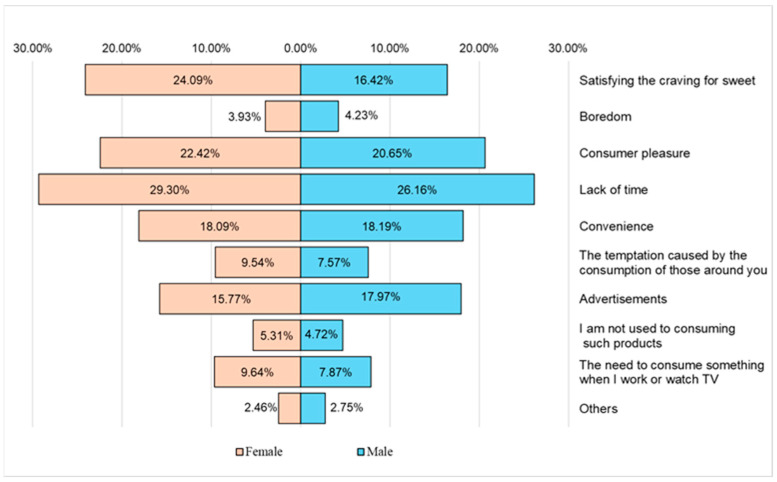
The main reasons for the consumption of fast food or ready-to-eat packaged food.

The main reasons indicated by the respondents regarding the consumption of junk food products were lack of time, satisfying the sweet tooth, the pleasure of consumption, and advertising (Figure 13).

Among the young respondents participating in this study, the processing of the data collected with the help of the questionnaire indicated increased preferences and addictions to the following fast food products: fried potatoes, hamburgers, shawarma, and caloric and high-fat food products (Table 2). French fries are often eaten together with hamburgers, which leads to an increased intake of calories and fats, and if they are also accompanied by sweetened drinks, the caloric intake becomes excessive in cases in which physical activity is reduced, and the associated consumption is frequent. In the long term, it can lead to excess weight, the accumulation of adipose tissue, increased insulin resistance, and the risk of metabolic syndrome. It is also known that junk food products contain numerous additives, including taste enhancers that stimulate consumption and create addiction (sodium mono-glutamate, E621).

## 4. Discussion

Following the dissemination of the questionnaire, 1017 responses were collected (Table 1), with 46.2% of respondents being male and 53.8% female. The respondents were classified into two age categories, namely teenagers (those aged between 16 and 18 years (*p* < 0.028), with 610 participants (i.e., 60%)) and young people aged between 19 and 25 years, with 407 participants (i.e., 40%). Most of the young people came from an urban environment (81.3%, *p* = 0.002) and were teenagers.

Among the young participants in this study, 12.7% (*p* = 0.613) declared that they practiced sports for at least an hour every day, 27% practiced sports for less than an hour every day, and 50% rarely engaged in sports activities, while 4.3% said that they did not usually practice sports. Thus, it can be observed there is an alarming trend toward sedentarism among the young participants in the study, with over 50% of them not performing enough exercise.

According to the respondents’ statements, 17.3% (*p* = 0.590) of them consumed alcoholic beverages very rarely or not at all, and 28.4% consumed 2–3 times a month, while the majority, 76.4%, consumed alcoholic beverages 2–3 times a week, and only 2.8% consumed one or more servings of alcoholic beverages daily.

Regarding body weight, most of the respondents were of normal weight (59.7% of them), especially female respondents (64.7% of them), compared to male respondents (53.8% of them). Among the 117 (11.5%) underweight respondents, most were female (74), while among the 188 overweight respondents (18.5%), most were male (111), and in the case of the 105 (10.3%) obese respondents, most were female (74).

In the consumption preferences of young people, meat and meat preparations predominated (37.07%), and among junk food products, pizza and pastry products were the main categories. Vegetable products accounted for 23.5% of daily consumption preferences, a percentage that is below the optimal level (over 50%) recommended by nutritionists (Figure 1).

The most preferred junk food products (Figure 2) by young people were fried potatoes (48.57%), pastry products (37.66%), snacks (33.04%), chips (28.52%), sweets (26.45%), and hamburgers (23.3%).

According to the statistically processed results, overweight and obese respondents frequently consumed french fries, hamburgers, hot dogs, pastries, shawarma, and packaged sandwiches, while normal-weight and underweight respondents more frequently consumed candies, packaged sweets, and chips (Figure 3).

Among the most consumed non-alcoholic drinks indicated by the respondents (Figure 4) were coffee (58.6%), sweetened non-carbonated drinks (37.17%), carbonated mineral water (33.82%), and natural juices (32.55%).

The statistical data indicate a tendency toward higher consumption of sweetened and energizing non-alcoholic drinks among overweight and obese young people (Figure 5), while normal-weight respondents generally consumed plain water, carbonated mineral water, and black tea. Those who were underweight consumed natural juices and lemonades.

It can be observed that an increased tendency to drink coffee is generally accompanied by smoking, and in the group of respondents, men were the ones with a greater tendency to smoke.

More than 28% of the young people interviewed could not answer if food exerted any impact on their health. We can appreciate that there is a need for an improvement in nutrition knowledge to understand the impact of food quality on health and to consciously choose nutritious foods (Figure 10).

A worrying fact found in the study was that although the respondents represented a young population, according to the recorded answers, there were a series of serious problems affecting their quality of life, mainly fatigue (44.64% of the respondents declared that they frequently felt tired) and states of nervousness (33.88% of respondents), and a higher percentage of these states were recorded among male respondents (Figure 11). Female respondents additionally faced, in a much higher proportion than male respondents, problems related to excessive emotional eating, depressive states, anxiety, panic attacks, or even lack of appetite. A possible explanation would be not only the excessive consumption of coffee and the relatively frequent consumption of alcoholic beverages or various junk food products but also the tendency toward sedentarism. Obviously, there are other factors that should be evaluated, including the duration of sleep, its quality, drug use, and exposure to pollutants, but diet, along with physical activity, plays a fairly important role in well-being. Moreover, the percentage of respondents who declared that they felt they developed an addiction related to the consumption of certain junk food products is worrying, namely coffee (50.48%), fried potatoes (38.9%), hamburgers (37.05%), shawarma (31.65%), and snacks (30.08%). Many of these products are rich in calories, saturated fat, and even trans fat (Figure 12). 

The consumption of fast food or ready-to-eat packaged food products was mainly determined by the lack of time and resorting to these fast food methods (55.46%); the pleasure of consumption (43.07%), which could also be correlated with food addiction; the satisfaction of sweet cravings (40.51%), which was much more pronounced in female respondents and also linked to food addiction; and advertising (33.74%), with female respondents more receptive to this than their male counterparts (Figure 13).

Regarding the influence of advertising on consumption decisions, the respondents declared that the most important ones were those broadcast by TV programs and those on billboards in public spaces.

Regarding consumption preferences for junk food products and alcoholic or non-alcoholic beverages, there are recent studies involving the Romanian population over 18 years of age that indicate, by comparison, that, in general, among young people (age segment 16–25 years), there is a significantly higher trend for the consumption of alcoholic drinks, energy drinks, and fast food products (especially fries, hamburgers, and shawarma) than among those over 30 years of age [50,51]. Also, these studies indicate a rather low trend of consumption of vegetable products (vegetables and fruits) among the population over 18 years of age, as well as a trend of insufficient hydration. These results are worrying and indicate the need for the involvement of medical specialists and nutritionists, as well as competent authorities in developing strategies to improve the eating habits and lifestyle of the population.

According to an official report published in 2020 by the National Institute of Public Health in Romania, generated following a study carried out at the national level, the percentage of smokers among teenagers and young people in the 15–24 age group is 19.97%, and 10.16% of those smoke daily, and 9.81% smoke occasionally [47]. The report also stated that the share of 15–24-year-olds who consumed alcohol in the last 12 months prior to the interview was 47.11%, and the share of respondents who did not consume alcohol at all was 38.15%, followed by 17.71%, accounting for those who consumed less than once a month. 

The WHO–HBSC 2017/2018 report states that Romania ranks 6th out of 44 countries, in descending order, in terms of the frequency of 15-year-old students who smoke at least once a week, and it also states that Romania is among the countries with an increased frequency of 15-year-old students who have consumed alcohol at least once in the last 30 days, so Romania is among the countries with consumption higher than 10% for boys compared to girls, along with Albania, Armenia, Georgia, and the Republic of Moldova [58].

Related to physical activity, the report published by the National Institute of Public Health in Romania indicates that the share of children and young people who, in a typical week, perform sports, fitness, or various recreational physical activities during their free time for at least 10 min continuously is higher in the 5–14-year age group (50.24%) and decreases to more than half of the value in the 15–24-year age group (20.47%), and the average time allocated to sports also decreases [47].

In a long-term study (2001–2018) among the American population, which included 29,970 children (aged 2–19 years) and 44,501 adults (aged ≥20 years), an increasing trend of consumption of junk food products was found among children and adults, especially among non-Hispanic white and black Americans compared with Mexican Americans [59].

A study published in 2022 conducted on the basis of a questionnaire completed by 355 respondents from the Kingdom of Saudi Arabia aged between 20 and 40 years highlighted an increased preference for the consumption of the following junk food products: french fries, shawarma, burger, and pizza [60].

Another recently published study (2022) draws attention to the increased tendency toward physical inactivity and the consumption of unhealthy foods, including junk food products, among school children from the southern region of India, and the consumption of unhealthy products was stimulated by taste, increased online food delivery, and attractive advertisements [61].

The main limitation of the present study is the low participation of young respondents from rural areas. Another limitation of the study is the subjectivity of the answers to the questions related to the perception of the impact of food on health because the answers are also influenced by the nutritional knowledge of the respondents.

## 5. Conclusions

The present study highlights a series of unhealthy trends in the eating and lifestyle habits of the young population, such as a tendency toward sedentarism; a trend of increased consumption of meat and meat preparations; and an increased preference for some junk food products such as fried potatoes, pastries, snacks, chips, hamburgers, sweetened soft drinks, coffee and even a relatively high consumption of alcoholic drinks. Many junk food products are caloric products, rich in unhealthy fats (saturated or trans), and present a risk of developing addiction to consumption, which is very dangerous for the body. In the long term, nutritional imbalances can lead to a risk of metabolic syndrome and even cardiovascular diseases. It is important to present and promote healthy alternatives to favorite junk food products, not only to young people but also to the population in general. Nutritional education and guiding young people to strengthen healthy habits are key to a sustainable increase in the quality of life and viable prevention in terms of pathologies in the field of metabolic disorders.

## Figures and Tables

**Table 1:** Socio-demographic and anthropometric characteristics, physical activity, and alcohol consumption of the respondents (n = 1017).

	Total Population n (%)	Male n (%)	Female n (%)
	1017 (100)	470 (46.2)	547 (53.8)
**Age (years)**		*p* = 0.028	
**16–18**	610 (60.0)	299 (63.6)	311 (56.9)
**19–25**	407 (40.0)	171 (36.4)	236 (43.1)
**Residence area**		*p* = 0.002	
Urban area	827 (81.3)	401 (85.3)	426 (77.9)
Rural area	190 (18.7)	69 (14.7)	121 (22.1)
**Level of physical activity/sport**		*p* = 0.613	
No physical activity	44 (4.3)	24 (5.1)	20 (4.2)
Yes, very rarely	509 (50.0)	239 (50.9)	270 (49.4)
Yes, 2–3 times a week	60 (5.9)	26 (5.5)	34 (6.2)
Yes, every day under an hour	275 (27.0)	119 (25.3)	156 (28.5)
Yes, daily for at least an hour	129 (12.7)	62 (13.2)	67 (12.2)
**Alcohol consumption (1 glass of wine, beer = 125 mL, 1 glass of drink pure alcohol = 50 mL)**		*p* = 0.590	
Daily more than one serving	14 (1.4)	8 (1.7)	6 (1.1)
Daily one serving	14 (1.4)	5 (1.1)	9 (1.6)
2–3 times a week	777 (76.4)	368 (78.3)	409 (74.8)
Once a week	13 (1.3)	6 (1.3)	7 (1.2)
2–3 times a month	488 (28.4)	86 (23.1)	402 (29.9)
Very rarely or not at all	176 (17.3)	72 (15.3)	104 (19.0)
**Body mass index (BMI)**		*p* < 0.0001	
Underweight (<18.5)	117 (11.5)	43 (9.1)	74 (13.5)
Normal weight (18.5–24.9)	607 (59.7)	253 (53.8)	354 (64.7)
Overweight (25–29.9)	188 (18.5)	111 (23.6)	77 (14.1)
Obese (≥30)	105 (10.3)	43 (9.1)	74 (13.5)

**Table 2:** The nutritional characteristics of the fast food products most consumed by young people.

Fast Food Product	Kcal	Saturated Fats	Trans Fats	Reference
Hamburger	297 (100 g)	4.49 g (100 g)	0.514 g (100 g)	[55]
Shawarma	357 (100 g)	7.14 g (100 g)	0 g (100 g)	[56]
Potatoes fried in vegetable oil	312 (100 g)	2.34 g (100 g)	0.06 g (100 g)	[57]

## Data Availability

The original contributions presented in the study are included in the article, further inquiries can be directed to the corresponding author.

## Supplementary Materials

The following supporting information can be downloaded at: https://www.mdpi.com/article/10.3390/nu16111769/s1, Survey: Questionnaire for evaluation of the junk food products consumption among the teenagers and young population from Romania.
