# Supplementary material for: Evaluation of the Consumption of Junk Food Products and Lifestyle among Teenagers and Young Population from Romania
Source: Nutrients. 2024 Jun 5;16(11):1769. doi: 10.3390/nu16111769 (PMC11174916; doi:10.3390/nu16111769)
Supplement: Supplementary file 1 [file nutrients-16-01769-s001.zip › nutrients-3030782-supplementary.pdf]

Questionnaire for Evaluation of the Junk Food Products Consumption among the Teenagers and Young Population from Romania

Personal data
1. Please mention your age (in years):
2. Please mention your sex:

- Masculine
- Feminine
- Others

3. Please indicate the geographical region where you live?

- Banat
- Bukovina
- Crişana
- Dobrogea
- Maramureş County
- Moldova
- Muntenia
- Oltenia
- Transylvania

4. Please indicate where you currently reside:

- Town
- Commune/Village

5. Please mention the level of education:

- General/primary education (without a baccalaureate degree)
- Secondary education (baccalaureate diploma)
- Post-secondary studies
- Higher education (bachelor's degree)
- Postgraduate studies (master, residency, doctorate, other specializations)

Anthropometric data
6. Please mention your weight (in kg):
7. Please mention your height (in cm):

8. Which of the following foods do you eat frequently? (multiple variants)

- ☐ Hamburgers
- ☐ Hot dog
- ☐ French fries
- ☐ Shaorma
- ☐ Packed sandwiches
- ☐ Chips
- ☐ Snacks
- ☐ Patisserie products, pastry
- ☐ Packaged cakes
- ☐ Candy
- ☐ Ice cream
- ☐ Other packaged sweet products
- ☐ Chewing gum
- ☐ Sweetened carbonated drinks
- ☐ Sweetened non-carbonated drinks
- ☐ Energy drinks
- ☐ Coffee
- ☐ I do not frequently consume any of the products

9. Which of the following foods do you consume the rarest? (multiple variants)

- ☐ Hamburgers
- ☐ Hot dog
- ☐ French fries
- ☐ Shaorma
- ☐ Packed sandwiches
- ☐ Chips
- ☐ Snacks
- ☐ Patisserie products, pastry
- ☐ Packaged cakes
- ☐ Candy
- ☐ Ice cream
- ☐ Other packaged sweet products
- ☐ Chewing gum
- ☐ Sweetened carbonated drinks
- ☐ Sweetened non-carbonated drinks
- ☐ Energy drinks
- ☐ Coffee

- None between products

10. Which of the following alimentation products have you never consumed? (multiple variants)

- Hamburgers
- Hot dog
- French fries
- Shaorma
- Packed sandwiches
- Chips
- Snacksuri
- Patisserie products, pastry
- Packaged cakes
- Candy
- Ice cream
- Other packaged sweet products
- Chewing gum
- Sweetened carbonated drinks
- Sweetened non-carbonated drinks
- Energy drinks
- Coffee
- None of the products

11. How often do you eat fast food or ready-to-eat packaged food? (only one option)

- Daily
- 2-3 times a week
- Once a week
- 2-3 times a month
- Very rarely or not at all

12. What is the reason you eat fast food or ready-to-eat packaged food? (several variants)

- Lack of time
- Consumer pleasure
- Convenience
- The temptation caused by the consumption of those around you
- Advertisements
- Boredom
- Satisfying the craving for sweet
- The need to consume something when I work or watch TV
- Others

- I am not used to consuming such products

13. Do you consider fast food products or packaged snacks to be healthy foods? (only one option)

- Yes
- Not
- I don't know

14. How many servings of vegetables (approx. 100 g) do you consume every day? (single variant)

- Very rarely or not at all
- One
- Two
- Three
- More than three

15. How many servings of fruit (approx. 100 g) do you consume every day? (single variant)

- Very rarely or not at all
- One
- Two
- Three
- More than three

16. How often do you eat meat? (single variant)

- Very rarely or not at all
- 2-3 times a month
- Once a week
- 2-3 times a week
- Daily

17. How often do you consume carbonated or sweetened drinks (1 serving = 330 mL, one glass)? (single variant)

- Very rarely or not at all
- 2-3 times a month
- Once a week
- 2-3 times a week
- Daily more than one serving
- Daily one serving

18. How often do you consume alcoholic beverages (1 glass of wine = 125mL, 1 glass of drink *pure alchool =50mL)? (single variant)

- Very rarely or not at all
- 2-3 times a month
- Once a week
- 2-3 times a week
- Daily more than one serving
- Daily one serving

19. How often do you consume fish or seafood? (single variant)

- Very rarely or not at all
- 2-3 times a month
- Once a week
- 2-3 times a week
- Daily

20. How often do you consume sweets / pastries? (single variant)

- Very rarely or not at all
- 2-3 times a month
- Once a week
- 2-3 times a week
- Daily

21. How often do you consume pasta, rice or other cereals? (single variant)

- Very rarely or not at all
- 2-3 times a month
- Once a week
- 2-3 times a week
- Daily

22. How often do you consume dairy products? (single variant)

- Very rarely or not at all
- 2-3 times a month
- Once a week
- 2-3 times a week

- Daily

23. How many eggs do you eat per week? (single variant)

- Very rarely or not at all
- 1 - 2 eggs
- 3 - 4 eggs
- 5 - 7 eggs
- More than 7 eggs

24. What category of food do you consume most often? (single variant)

- Fast food products
- Pizza, snacks, pastries, sweets
- Products made from sausages and preserves
- Food cooked in restaurants
- Home-cooked food

25. What type of cooked foods do you eat most often? (single variant)

- Fried foods
- Food prepared at the henhouse on wood or coal
- Grilled food
- Food prepared in the oven
- Boiled or steamed foods
- Thermally unprocessed food
- Food cooked under vacuum
- Others

26. How much water do you drink per day? (single variant)

- Less than 1 L
- 1 l
- 2 l
- 3 l
- Over 3 L

27. What category of liquids are you used to consuming most often? (single variant)

- Alcoholic beverages: sparkling drinks / wine, beer, etc.
- Carbonated or sweetened soft drinks including tonic ones
- Coffee

- Tea
- Natural juices
- Drinking water (from tap, well)
- Still mineral water
- Carbonated mineral water
- Other type of water

28. What category of food predominates in the daily diet? (single variant)

- Vegetables and fruits
- Cereals and pasta
- Dairy
- Fish and seafood dishes
- Meat
- Eggs
- Prepaired from meat (sausages, minced meats, canned food, etc.)
- Pizza, pitchers and pastries
- High-fat foods
- Fast food products

29. Do you usually do sports / exercise? (single variant)

- Not
- Yes, very rarely
- Yes, 2-3 times a week
- Yes, every day under an hour
- Yes, daily for at least an hour

30. Where do you do sports/movement? (multiple variants)

- At home
- Outdoors
- Gym
- I don't do sports/movement

31. Do you smoke? (single variant)

- Yes, excessive daily
- Yes, 1-2 cigarettes daily
- Yes, 2-3 times a week
- Yes, occasionally

- Not

32. How often do you consume coffee? (one variant)

- Daily
- 2-3 times a week
- Once a week
- 2-3 times a month
- Very rarely or not at all

33. How much coffee do you consume daily? (one variant)

- A cup
- 2-3 cups
- 4-5 cups
- More than 5 cups
- Rarely or not at all consumption

34. What type of problems do you encounter? (multiple variants)

- I'm frequently tired
- I'm frequently nervous
- I'm frequently depressed
- I frequently have panic attacks/anxiety states
- I'm frequently agitated
- I frequently have palpitations
- I frequently have migraines
- I eat emotionally, excessively
- I have no appetite
- I'm fine, I have no problem

35. Do you think you have an addiction related to the consumption of certain fast food or **ready-to-eat packaged foods? (single variant)**

- Yes
- Not
- I don't know

36. On which types of food do you consider that you have developed an addiction? (multiple variants)

- ☐ Hamburgers
- ☐ Hot dog
- ☐ French fries
- ☐ Shaorma
- ☐ Packed sandwiches
- ☐ Chips
- ☐ Snacks
- ☐ Patisserie products, pastry
- ☐ Packaged cakes
- ☐ Candy
- ☐ Ice cream
- ☐ Other packaged sweet products
- ☐ Chewing gum
- ☐ Sweetened carbonated drinks
- ☐ Sweetened non-carbonated drinks
- ☐ Energy drinks
- ☐ Coffee
- ☐ None of the variants
